# II.1.5 Phenotyping pearl millet for adaptation to drought

**DOI:** 10.3389/fphys.2012.00386

**Published:** 2012-10-19

**Authors:** Vincent Vadez, Tom Hash, Francis R. Bidinger, Jana Kholova

**Affiliations:** GT-1 Biotechnology, ICRISATPatancheru, India

**Keywords:** drought, pearl millet

## Abstract

Pearl millet is highly resilient to some of the driest areas of the world, like the Sahel area or fringes of the Thar desert in India. Despite this, there is a wealth of variation in pearl millet genotypes for their adaptation to drought and the object of this paper was to review some related work in the past 25 years to harness these capacities toward the breeding of better adapted cultivars. Work on short duration cultivars has been a major effort. Pearl millet has also some development plasticity thanks to a high tillering ability, which allows compensating for possible drought-related failure of the main culm under intermittent drought. The development of molecular tools for breeding has made great progress in the last 10–15 years and markers, maps, EST libraries, BACs are now available and a number of quantitative trait loci (QTLs) for different traits, including drought, have been identified. Most of the work on drought has focused on the drought tolerance index (DTI), an index that reflect the genetic differences in drought adaptation that are independent of flowering time and yield potential. The DTI is closely associated to the panicle harvest index (PNHI), a trait that relates to a better grain setting and grain filling capacity. Initial work on the DTI involved empirical breeding and selection based on PNHI. A QTL for PNHI has then been identified and introgressed by marker-assisted backcrossing. More recently, a thorough dissection of that QTL has been carried out and shows that high PNHI is related to the constitutive ability of tolerant lines to save water (lower leaf conductance and sensitivity of transpiration to high vapor pressure deficit) at a vegetative stage and use it for the grain filling period. However, there is no contribution of root traits in this QTL. Current work is taking place to map these water saving traits, understand their genetic interactions, and design ideotypes having specific genetic make-up toward adaptation to specific rainfall environments.

## General information

### Importance of pearl millet in the human diet

Pearl millet (*Pennisetum glaucum* (L) R Br) is a hardy cereal crop, grown mostly in marginal environments in the arid and semi-arid tropical regions of Asia and Africa. It is grown primarily for grain production but is also valued for its fodder, the importance of which has been rising in recent years. Pearl millet is grown in areas with very limited rainfall (300–500 mm in the majority of cases), where crops such as maize or sorghum are very likely to fail in most years. Therefore, pearl millet is a central component of the food security of the rural poor in dry areas.

With regard to nutritional quality, pearl millet is at least equivalent to maize and generally superior to sorghum in protein content and quality, protein efficiency ratio (PER) values, and metabolizable energy levels. Pearl millet does not contain any condensed polyphenols such as the tannins in sorghum that can decrease digestibility. It is deficient in essential amino acids, although it contains 35% more lysine than sorghum (Rooney and McDonough, 1987). Pearl millet grain contains 5–6% oil (Jambunathan and Subramanian, 1988) and is also rich in important micronutrients such as iron and zinc. Moreover, among all cereals, it is the cheapest source of energy, protein, iron, and zinc. These qualities make pearl millet the major contributor to protein, iron, and zinc intake in the regions where it is grown, accounting, for example, for 20–30% of the zinc intake, and 35–50% of the total iron intake of low-income consumers.

Yet, pearl millet remains a food for the poor and is stigmatized by its frequent association with poverty. As a result, the consumer choice is to move away from pearl millet consumption whenever possible. In India, for example, the food use of coarse cereals has been declining during the last two to three decades, owing to a shift in consumption to fine cereals such as rice and wheat. Pearl millet is no exception to this; its consumption per capita and per year in India in 1999–2000 was only 3.7 kg out of 147 kg for all cereals. Despite the decline in overall per capita consumption of pearl millet, it remains an important staple in producing regions, with 66.7 kg per capita consumed in Western Rajasthan, and 62.6 kg in Gujarat. However, even though pearl millet remains at the heart of food security in large areas of the semi-arid tropics, there is a need to diversify its uses, in particular commercially, to make it more attractive and fully use its potential for these regions.

Alternative uses of pearl millet such as for poultry feed are on the increase. Indeed, Smith et al. (1989) report that pearl millet can replace maize in chick diets without affecting weight gain or feed efficiency. The crop residue/straw of dual-purpose pearl millet is an important source of fodder, accounting for 40–50% of dry matter intake year round, and the only source of feed in the dry months. The use of pearl millet for fodder predominates in low input crop-livestock systems and is likely to become a very important component of the sustainability of such systems. In fact, the growing demand for milk and meat is reflected in the rising price of straw of cereals like pearl millet (Hash et al., 2003).

### Cultivated area and yield performance under optimal conditions

The total area cultivated with pearl millet worldwide is 26 million ha, comprising ca 11 million ha in each of West Africa and South Asia, and ca 2 million ha in each of East Africa, Southern Africa, and Brazil [International Crops Research Institute for the Semi-arid Tropics (ICRISAT)] and Food and Agriculture Organization of the United Nations, (FAO, 1996). India is the largest producer, with 9–10 million ha in area and 7–8 million tons of grain production. Pearl millet is cultivated in the hot dry parts of India in regions receiving low annual rainfall ranging from 300 to 800 mm. Between 1970 and 2001, the area under the crop in India declined from 12.1 to 9.4 million ha but production increased from 5.7 to 6.9 million tons due to an increase in yield from 473 to 740 kg ha^−1^. Pakistan has ca 500,000 ha cultivated to pearl millet. In Africa, the largest pearl millet growing countries are Senegal, Mali, Burkina Faso, Niger, Nigeria, Chad, and Sudan. In West and Central Africa, open-pollinated varieties are cultivated on 16 million ha, with a production of 11.5 million tons and productivity of 800 kg ha^−1^.

The growth potential of any crop species is a function of its growth rate and the length of the growth cycle. This is obviously conditioned by the agronomic potential where it is grown (relating to water, light, and nutrients). In general, pearl millet is rarely grown in areas enjoying high agronomic potential. It is almost invariably grown in low rainfall areas (van Oosterom et al., 1996a; Van Oosterom et al., 1996b) and under marginal fertility which, in fact, results in an incomplete use of the available water (Payne et al., 1990). Thus, environmental factors are usually the main limitations to its growth potential. Even under favorable conditions, pearl millet tends to have a shorter crop cycle than other cereals because it has a “built in” drought escape mechanism (early flowering) inherited from its wild progenitors, having evolved in semi-desert environments with adapted short life cycles. Therefore, pearl millet is short cycled, has a short grain-filling period and has small seed sizes. Its growth potential is no match for other longer-duration cereals growing in favorable environments. Yet, it enjoys a high crop growth rate that confers a fairly high growth potential under optimal conditions (Begg, 1965), relating in particular to its being a C_4_ plant, with a large leaf area index (LAI) due to its erect type (Craufurd and Bidinger, 1989), and high radiation-use efficiency (RUE; Squire et al., 1986). The maximum RUE recorded ranges between 2.5 g MJ^−1^ (Squire et al., 1986) and 4.0 g MJ^−1^ (Ram et al., 1999), although most data range between 1.0 and 2.0 g MJ^−1^. One limitation to RUE is early in the crop cycle, when the LAI is low. There seems to be genetic variation in the rate of leaf appearance, probably because of differences in the base temperature, although this has not been exploited in breeding (Bidinger and Hash, 2003).

Landrace open-pollinated cultivars of pearl millet usually exhibit high levels of vegetative vigor and very high biomass production. However, the harvest index (HI) of these traditionally tall cultivars is only 15–20%. This is largely due to the fact that the photoperiod-mediated change in the total growth duration mostly affects the length of the vegetative period (Carberry and Campbell, 1985). It has been reported that a crop of a local variety of pearl millet, cv Ex-Bornu, grown in Northern Nigeria under high fertility conditions without irrigation, could produce 22 tons ha^−1^ of above ground dry matter 90 days after sowing, although only 3.2 tons of this (14.5%) was grain (Kassam and Kowal, 1975). In contrast, grain yield on a field basis of over 5 tons ha^−1^ was produced by semi-dwarf hybrids maturing in 85 days in India (Rachie and Majmudar, 1980). Experimental yields of up to 8 tons ha^−1^ have even been reported (Burton et al., 1972).

### Genetic and genomic resources

Over the past decade, ICRISAT and its partners have made substantial investments in developing mapping populations (Hash and Witcombe, 1994) and in DNA-based molecular marker systems including restriction fragment length polymorphism (RFLP; Liu et al., 1994), sequence-tagged sites (STS; Devos et al., 1995), amplified fragment length polymorphism (AFLP), and simple sequence repeat (SSR) markers (Qi et al., 2000; Allouis et al., 2001), and a bacterial artificial chromosome (BAC) library (Allouis et al., 2001) for pearl millet. These genetic tools have been used to develop a DNA marker-based linkage map for pearl millet (Liu et al., 1994), and to map quantitative trait loci (QTLs) conferring resistance to biotic stresses (Jones et al., 1995, 2002; Morgan et al., 1998) and tolerance to terminal drought stress (Yadav et al., 2002b). They have also been used for: (1) identification of QTLs for flowering time, that appear to be largely responsible for genotype-by-environment interaction (GEI) for grain and stover yield under favorable growing conditions (Yadav et al., 2002a); (2) diversity assessment (Liu et al., 1992; Bhattacharjee et al., 2002); (3) studies of recombination rates (Busso et al., 1995; Liu et al., 1996); (4) analysis of the domestication syndrome (Poncet et al., 2000, 2002); and (5) comparative genomics (Devos et al., 1998; Devos and Gale, 2000).

Levels of DNA marker polymorphism in pearl millet are very high, even between elite inbred parental lines of hybrids adapted to growth in India. The current pearl millet DNA marker-based genetic linkage map covers about 700 cM (Haldane function) distributed across the expected seven linkage groups for this diploid (2*n* = 2*x* = 14) species, and at least one free-floating pair of linked RFLP markers. However, telomeric regions capping the chromosomes have not yet been mapped (Devos, pers. communication). These DNA marker-based linkage groups have not been definitively linked with the chromosome map of this species (Minocha and Sidhu, 1981; Kaul and Sidhu, 1997), which has been developed over the past 35 years using morphological markers (Anand Kumar and Andrews, 1993) and conventional cytogenetic methods (Jauhar and Hanna, 1998).

Compared to most other grasses, the pearl millet genome appears to have undergone a large number of structural re-arrangements (Devos and Gale, 2000). It seems likely that these re-arrangements could have been associated with the evolution and maintenance of adaptive gene complexes that permit this highly cross-pollinated crop and its wild progenitors to thrive in environments where they are routinely subject to severe abiotic stresses (e.g., seedling and reproductive heat stress, sand blasting of seedlings, soil nutrient deficiencies and soil toxicities, drought stress). These structural re-arrangements continue to be common in pearl millet, although marker relationships are nearly all colinear across the 10 pearl millet mapping population skeletons mapped to date (Liu et al., 1994, 1996; Devos and Gale, 2000; Azhaguvel, 2001; Kolesnikova, 2001).

Several pearl millet mapping populations of moderate size (120–275 progenies) have been developed at ICRISAT Headquarters at Patancheru, India as sets of F_4_ progeny bulks and their F_3_ test-crosses, derived from individual skeleton-mapped F_2_ plants (Hash and Witcombe, 1994; Hash and Bramel-Cox, 2000). These now involve some 10 pairs of genetically diverse inbred lines of Asian, African, and American origin, selected for QTL mapping of disease resistances (Jones et al., 1995, 2002), abiotic stress tolerances (Howarth et al., 1997; Yadav and Weltzien, 1999; Yadav et al., 1999, 2000, 2002b), grain and stover yield and quality components (Yadav et al., 2002a), and morphological markers (Azhaguvel, 2001). Several of these populations have parents of contrasting Indian and West African origin (e.g., PT 732B × P 1449-2; H 77/833-2 × PRLT 2/89-33; ICMB 841 × 863B; and W 504 × P 310-17) that are expected to differ for many traits.

Being domesticated from wild relatives, i.e., *Pennisetum fallax* and *Pennisetum violaceum* (Stapf and Hubbard, 1934), later reclassified as *P glaucum* (de Wet et al., 1992), living in the southern fringes of the Sahara, pearl millet has a number of characteristics that confer upon it adaptation to drought conditions. The different characteristics and whether these have been exploited for breeding purpose are discussed below.

#### Tillering and developmental plasticity

This is an attribute that derives from wild progenitors. Pearl millet develops primary tillers, and then secondary tillers from the primary ones, about every 45–50°C days (base temperature of 10°C). Because of this high tillering ability and because the length of the period between floral initiation and flowering is similar, plants have tillers at all stages of apical development at all times (Craufurd and Bidinger, 1988a,b). This developmental plasticity allows pearl millet to compensate for potential failure of the main and primary tillers in the case of a mid-season drought. The secondary tillers would, to a large extent, compensate for the yield loss on the main tillers by a larger number of them developing a panicle, as long as the relief from mid-season drought makes sufficient water available for the secondary tillers to reach maturity (Mahalakshmi and Bidinger, 1986). Because of this plasticity, it is often considered that pearl millet is not affected very much by mid-season drought, provided that moisture is available for the end of the season (Mahalakshmi and Bidinger, 1985a,b).

#### Flowering time

In most crops, matching plant phenology with the stress environment is a key factor in adaptation to drought. Flowering time, a so-called “drought escape mechanism,” is the major component of pearl millet's adaptation to water-scarce environments (e.g., Bidinger et al., 1987a,b; see “Drought Resistance Index” below). The floral morphogenesis stage, GC2, which is the period between floral initiation and flowering, appears to be fairly constant across genotypes of pearl millet. The relative shortness of that period (about 350°C-days (degree-days, which represent a thermal unit of temperature accumulation above a baseline temperature of 10°C for pearl millet—for instance 1 day with a mean temperature of 25° would accumulate 25 – 10 = 15°C-days) allows pearl millet to complete it with relatively limited water (Dancette, 1983). Therefore, earliness is an important drought escape attribute of pearl millet and is, indeed, a major component of GEI. For instance, in the case where the rains stop early, a 1-week difference in the time to flowering between two genotypes brings about a 30% reduction in the grain-filling period and gives the early cultivar more chance to escape drought stress, whereas the late cultivar is likely to suffer the stress before or during reproduction. However, it appears that the prospect of breeding for earliness is limited because of the often poor predictability of rainfall events in the semi-arid tropics. Therefore, there seems to be an optimal time for flowering, suited to the average season length. It is within that particular range of flowering times for any particular environment that other traits likely to improve performance under water-limited conditions must be found.

In West Africa, the sensitivity of pearl millet to the photoperiod (Clerget et al., 2004) is a way that it has evolved to “trigger” an escape mechanism, since it appears that the timing of flowering is closely related to the end of the rainy season. In other words, pearl millet flowers “on time” to ensure that it can complete its maturation cycle with the remaining soil moisture (Kouressy et al., 1998). Any genotype with delayed flowering may be exposed to serious stress conditions during its reproduction phase.

#### Drought resistance index

It has been found that about 50% of yield variation under drought stress conditions could be explained by differences in the yield potential of genotypes and their flowering time (Bidinger et al., 1982, 1987a). Therefore, data on yield under stress conditions would have little relation to drought tolerance per se without removing the components that are explained by yield potential and phenology. This led Bidinger et al. (1987b) to develop an index, the “drought resistance index” (DRI), in which the effect of yield potential and drought escape (flowering time) are removed by assuming that yield under stress is a function of yield potential (control yield in the test environment), drought escape (proxied by time to flowering), and a residual that accounts for drought tolerance/susceptibility. So that:

$$\hat{\text{Y}}\text{s}=\text{aYc}+\text{bFl}+\text{Residual}$$

where Ŷs is the predicted yield under stress based on the yield under control conditions, respectively, the flowering time (Fl) and a residual. This residual variation in grain yield under stress that is not explained by either the potential yield (Yc) or by the flowering time (Fl) represents the DRI. The value of the residual (= DRI) is obtained as follows: DRI = Ys – Ŷs, where Ys is the actual grain yield under stress conditions. Therefore, the DRI represent the deviation in grain yield under stress from a baseline yield that depends on the yield potential and flowering time and it therefore allows to compare genotype's performance regardless of their yield potential and flowering time.

A similar approach has been used in other stresses, for example, to separate salinity tolerance per se from yield potential in a set of chickpea germplasm lines (Vadez et al., 2007). The DRI approach has been used in a selection programme for improved drought tolerance (see below), using the panicle harvest index (PNHI), i.e., the ratio of grain yield to panicle yield on a plot basis, as a proxy to assess the DRI.

#### Rooting ability

Pearl millet is known to be deep and profusely rooted, with the ability to match its rooting to water availability in a very plastic manner, leading to a highly varying root growth to shoot growth ratio, depending on the intensity of water limitation (Squire et al., 1987). During the vegetative period, root growth is very profuse, but little is known about root growth during the post-anthesis period, although it has been reported that it continues well into grain-filling in long-duration West African cultivars (Do et al., 1989). Root penetration rates between 3.5 and 4.5 cm day^−1^ have been reported in sandy soils (Chopart, 1983; Azam Ali et al., 1984). Root depth is dependent on the season length of the cultivar, and can be as deep as 3 m in long-duration varieties, in contrast to only 140 cm in short-duration cultivars (Chopart, 1983). Lateral root spreading is also a major feature of pearl millet, with the soil volume exploration at low planting density being as much as 6 m^3^ (Chopart, 1983).

It is often assumed that water uptake and, consequently, water limitation is what limits pearl millet production in a low rainfall environment. However, it has been shown that water may not be the most limiting factor, at least in the sandy soils of Niger, where substantial water storage and drainage have been found below the deep root zone (Payne et al., 1990). This may not be the case in all soils where pearl millet is grown. In fact, roots appear to play an important role in pearl millet genotypes that differ in the presence or absence of a major terminal drought tolerance QTL (Vadez et al., 2005). Further efforts are needed to clarify the extent of the role of the root in the drought tolerance of pearl millet.

#### Water-use efficiency

Being a C_4_ plant, pearl millet already has high transpiration efficiency (TE). However, it seems that the major strategy of pearl millet is to maximize carbon fixation as long as water is available. Therefore, stomatal movements adapt in such a way that the transpiration rate is kept as high as possible (Squire, 1979; Henson and Mahaklakshmi, 1985). It also appears that stomata are sensitive to the vapor pressure deficit (VPD), particularly during the pre-flowering stage, this being related to differences in the abscicic acid (ABA) content of the leaves (Henson and Mahaklakshmi, 1985). In any case, there have been no studies to assess the range of variation in TE across a diverse range of pearl millet cultivars and lines, nor on the sensitivity of stomata to VPD.

At the plot level, water-use efficiency (WUE) values of 300–400 kg biomass ha^−1^ cm^−1^ water have been reported, assuming a full ground cover (LAI > 3–4) (Singh and Singh, 1995). Under low planting density, the WUE usually drops to the range 50–150 kg ha^−1^ cm^−1^, mostly because of an increased evaporation component (Payne, 1997), itself high because of the fertility-related low sowing density. Therefore, it seems that fertility may be the number one factor to improve the WUE at the plot level.

#### QTL for terminal drought tolerance

In most of the environments where pearl millet is grown, the crop is facing stress during the grain-filling period, in particular in Northern India (van Oosterom et al., 1996a,b). Therefore, work has focused on identifying QTLs for terminal drought tolerance using the PNHI as a selection criterion.

## Methodology

### Breeding strategy

#### Possible definitions

The overall goal of a breeding programme for drought stress is, ultimately, an improved genetic yield, or a more stable yield, under drought conditions. These two objectives are not necessarily related. The latter, the stabilization of yield across environments in drought-prone areas, is very important because of the large differences in the coefficient of variation of pearl millet production at the all-India level (26%) compare to that in Rajasthan state (53%), which is characterized by very low and erratic rainfall. There are different ways to assess what is commonly called “drought tolerance,” and this depends mostly on how close the assessed trait/parameter is from the final target—an increased genetic yield. Therefore, the approaches to drought tolerance vary. Three categories can broadly be defined, with advantages and drawbacks as highlighted below:

- Drought tolerance is seen purely as a higher and more stable yield under drought conditions, which is fully in line with the ultimate goal. However, in almost all cases, this is related to a large GEI because yield is the integration of many different processes, each of them having a close interaction with the environment.
- Drought tolerance is considered as the maintenance of different development and growth processes, such as leaf expansion, at levels that are close to control well-watered plants. Here, we assume that these would remain well-linked to yield performance. This approach is straightforward and may be easier to capture than yield itself. However, some of these traits can be cumbersome to measure, which may not allow time to assess large numbers of accessions and progenies.
- Drought tolerance can be seen as more upstream, at the organ or cell level, and can be seen as the capacity to sustain certain biological mechanisms, such as maintaining leaf turgor, close to the level of well-watered plants. Measuring such traits requires screening under controlled environment conditions where better management and reproducibility of environmental variation can help reach low levels of GEI. However, the main drawback of this approach is that the traits may be loosely related to the final yield under stress.

#### Drought resistance index and its relationship to the panicle harvest index

Pearl millet is very resilient to intermittent drought because of its developmental plasticity and its capacity to compensate yield losses on the main tillers with grain production on secondary tillers. For these reasons, it is often considered that mid-season drought is a less important problem for pearl millet, and that tolerance to terminal drought affecting the plant during grain filling is the major target for drought improvement. It has been found that yield under stress is, in part, determined by the yield potential of the material tested plus some escape mechanisms related to its phenology. Bidinger et al. (1987a,b) have encapsulated drought tolerance per se from these non-stress related parameters into the DRI, through correlation analyses of yield data under stress with flowering time and yield under non-stressed conditions. The approach has been to work backwards from measured differences in grain yield in managed drought environments, to readily-measurable aspects of field performance that explain those differences (Fussell et al., 1987). From that point, various yield component parameters were measured under different watering regimes, using some pearl millet varieties differing in their tolerance to terminal drought (Table 1). This analysis revealed that the number of grains per panicle and the 100-grain weight were the yield components most affected under terminal drought conditions, leading to a decrease in the PNHI. The PNHI can also be called the threshing index and it represents the proportion of grain weight that a whole panicle contains. A high panicle index reflect that most florets of the panicle have successfully developed in a grain, and that this grain has filled up to its potential.

**Table 1:** **Consequences of different levels of terminal stress tolerance on pearl millet panicle components and panicle harvest index (PNHI; Source: hypothetical data extracted from Bidinger, 2002)**.

**Genotype level of tolerance**	**Panicle structural part (g)**	**Grains per panicle (no.)**	**Single grain mass (g)**	**Total grain mass (g)**	**Total panicle mass (g)**	**PNHI (%)**
Non-stress conditions	5.0	1500	0.0100	15.0	20.0	75
Escape: early flowering	5.0	1500	0.0085	12.8	17.8	72
Tolerant	5.0	1350 (−10%)	0.0085 (−15%)	11.5	16.5	70
Intermediate	5.0	1200 (−20%)	0.0070 (−30%)	8.4	13.4	63
Susceptible	5.0	1200 (−20%)	0.0050 (−50%)	6.0	11.0	55

DRI represents the share of the variation in yield across a set of genotypes that cannot be explained either by differences in yield potential or time to flowering, and is closely related to yield under stress conditions (Table 2). Bidinger et al. (1987a,b) have also shown DRI was closely related to the PNHI and therefore, a high DRI was closely related to a higher percentage grain set and better grain filling (Table 3), which are the major components of the PNHI. In subsequent works, the PNHI hasthen been used as an indirect proxy for DRI, and is readily and cost-effectively measured. PNHI is a particularly effective variable for post-flowering stress, because the mass of the structural parts of the panicle (which complete their growth prior to flowering) is largely unaffected by stress, whereas the mass of grain is significantly affected by both floret abortion and reduced grain filling (Bidinger and Mukuru, 1995; Table 1).

**Table 2:** **Relations between the drought resistance index (DRI) and various agronomic factors measured either under fully irrigated conditions (control) or under terminal drought stress (stress) [Adapted from Bidinger et al. (1987b)]**.

**DRI versus:**	**1981**	**1982**
**MID-SEASON STRESS**		
Control flowering	0.06	0.08
Control yield	0.06	0.06
Stress yield	0.67^***^	0.58^***^
Stress/control yield	0.47^***^	0.46^***^
**TERMINAL STRESS**		
Control flowering	0.00	−0.05
Control yield	0.05	0.05
Stress yield	0.55^***^	0.72^***^
Stress/control yield	0.55^***^	0.61^***^

Stress/control represents the ratio of the yields under each respective treatment. ^*^P < 0.05; ^**^P < 0.01; and

*** P < 0.0001.

**Table 3:** **Relationships between the drought resistance index (DRI) and yield and various yield components under a range of water stress regimes, i.e., a mid-season stress or a terminal water stress [Adapted from Bidinger et al. (1987b)]**.

**DRI versus:**	**1981**	**1982**
**MID-SEASON STRESS**		
Grain m^−2^	0.39^***^	0.49^***^
Plant m^−2^	0.03	0.28^*^
Panicle plant^−1^	0.08	−0.19
Grain panicle^−1^	0.26^*^	0.31^**^
Individual grain mass	0.10	0.32^**^
Panicle m^−2^	0.07	0.18
Grain yield panicle^−1^	0.24^*^	0.34^**^
**TERMINAL STRESS**		
Grain m^−2^	0.46^***^	0.45^***^
Plant m^−2^	−0.12	−0.0
Panicle plant^−1^	0.10	0.07
Grain panicle^−1^	0.53^***^	0.37^**^
Individual grain mass	0.25^*^	0.40^***^
Panicle m^−2^	0.10	0.06
Grain yield panicle^−1^	0.69^***^	0.58^***^

* P < 0.05;

** P < 0.01; and

*** P < 0.0001.

#### Trait-based conventional approach

This approach was initially used to select genotypes achieving a high PNHI under terminal drought conditions. PNHI was initially tested in hybrid parent breeding, where it was used as a selection criterion by the following procedure:

1. Conduct bidirectional selection for combining ability for high and low PNHI in replicated potential maintainer (B) and restorer (R) line test cross nurseries (three testers each) grown in managed terminal drought stress environments.
2. Cross parents selected for high and low PNHI under stress conditions on three different A or R line testers from those used in the original test cross nurseries in which selection was carried out.
3. Evaluate these test crosses for general combining ability (GCA) for PNHI, grain yield and yield components, in both fully irrigated control environments and in managed stress environments.

In both experiments, the differences between the high and low PNHI selections in the irrigated control environments were small and generally not statistically significant (1% for PNHI itself, 2% for grain yield, and 3% for seed mass). Differences in the terminal stress environment between the high and low selections were generally statistically significant and of a greater magnitude under stress conditions (Table 4). For example, the combining ability of high PNHI selections exceeded that of the low PNHI selections by approximately 5–8% for PNHI itself, by 9–13% for grain yield, and by 6–7% for seed mass. Thus, selection for or against GCA for PNHI under terminal stress had little effect on the combining ability of elite parental lines in non-stress conditions, but resulted in a significant difference in their combining ability for both PNHI itself and for grain yield under terminal stress.

**Table 4:** **Combining ability for PNHI, yield and yield components of restorer and maintainer lines selected for high (nine lines) and low (nine lines) combining ability for PNHI, in test cross nurseries grown under terminal drought stress at ICRISAT-Patancheru**.

	**PNHI (%)**	**Grain yield (g m^−2^)**	**Grain no (10^3^ m^−2^)**	**Seed mass (mg seed^−1^)**
**RESTORER LINES**				
High PNHI selections	64.8	218	31.1	6.86
Low PNHI selections	59.8	192	29.5	6.38
SED	0.4	2.7	3.7	0.69
**MAINTAINER LINES**				
High PNHI selections	63.6	189	29.7	6.31
Low PNHI selections	60.4	173	28.9	5.93
SED	0.4	2.8	3.9	0.57

Data are means of three test crosses per line and 3 years of replicated evaluations in managed terminal stress environments in the dry season at ICRISAT-Patancheru. [SED, standard error of the difference; Adapted from Bidinger et al. (2000)].

PNHI was also used as a selection criterion in open-pollinated variety breeding for improved tolerance to terminal stress, using S_1_ progeny selection in a random mating population (data not shown). The selection was based on PNHI under terminal stress (PNHI/stress) compared to two controls: selection on the basis of grain yield in a paired irrigated control environment (yield/control), and selection of random S_1_ progenies (random check). Two cycles of selection were conducted, using 810 S_1_ progenies from the parent population in cycle 1, and 400 S1 progenies from each of two subpopulations (formed from 50 progenies from the first cycle) representing the PNHI/stress and yield/control selection alternatives, in cycle 2. Overall, after two cycles of selection, selecting experimental varieties on the basis of composite progeny PNHI in terminal stress environments improved PNHI by 1–3% and grain yield by 2–8% under terminal stress (in comparison to control experimental varieties, based on randomly selected progenies).

#### Trait-based molecular breeding approach in current use

This is the current approach to pearl millet breeding for drought tolerance. It is based on the fact that PNHI remains a highly complex trait for which a molecular approach can increase precision during the selection process. For molecular breeding, the development of recombinant inbred lines (RILs) is needed to link phenotypic data and marker data, and potentially identify QTLs, i.e., genome portions that are related to phenotypic data. Prior to that, the parents used for crossing should comply with a number of characteristics to maximize the chances of discovering RILs. They should: (1) be chosen from large number of accessions; (2) have maximum phenotypic contrast; (3) have large genotypic contrast; and (4) be similar for certain phenotypic traits that can interact with the trait of interest (yield), such as time to flowering or photoperiod sensitivity.

Although parents chosen for crossing and development of RILs, may display large phenotypic contrast, they may have little DNA-level polymorphism. Such a situation limits the marker coverage that can be used to map the genomic portion responsible for the observed phenotypic differences. Having a limited number of polymorphic markers will, in most cases, increase the cost and time to get QTLs, and lower the resolution of the QTLs. An alternative in such cases is to develop different types of marker with a higher resolution, such as single nucleotide polymorphisms (SNPs). Finally, the crossing of parents may involve certain criteria that can have a strong influence on the response to drought or salinity. Indeed, we have shown earlier that the yield under terminal drought was a function of the yield potential under no stress, a drought escape mechanism, and DRI per se. Therefore, it not is advisable to cross parents with large variations in yield potential or flowering time if the intention is to develop a RIL population to map terminal drought tolerance.

**Figure 1:**
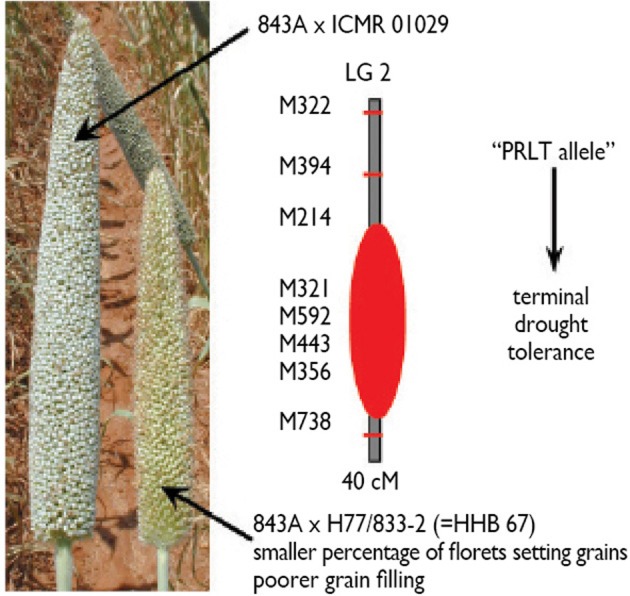
**Phenotypic expression of stress effects on the panicle of sensitive HHB67 (= 843A × H77/833-2) and an improved version of this hybrid (843A × ICMR 01029), and showing a better seed set and a better grain filling in the tolerant hybrid**. ICMR01029 is an introgression line with a terminal drought tolerance on linkage group 2 from donor parent PRLT/89-33, after four backcrosses using H77/833-2 (Source: Hash, unpublished).

This approach has been used successfully for the identification of terminal drought tolerance QTLs (Yadav et al., 2000, 2002b, 2003), and the introgression of a terminal drought tolerance QTL into the background of the popular pearl millet hybrid HHB67 to create the new hybrid HHB67-improved. This terminal drought tolerance QTL has a major effect, explaining over 30% of the yield variation under terminal drought. It is located on linkage group 2 (LG2) (Figure 1). Further efforts are still needed to reduce the size of that QTL to improve the precision of its introgression. Better marker coverage of the QTL region would be needed for that, and work toward that aim is in progress.

#### Scheme for RIL development and testcross hybrid testing

**Figure 2:**
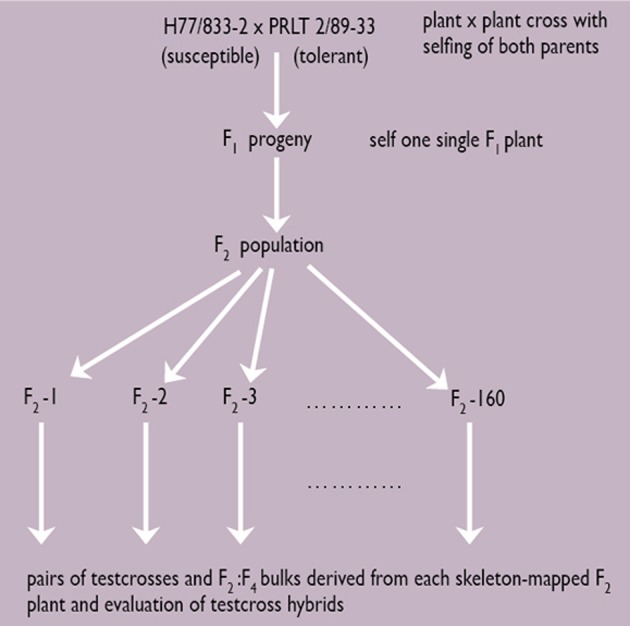
**Strategy for the development of a skeleton map and identification of drought tolerance QTLs (Source: Hash, unpublished)**.

**Figure 3:**
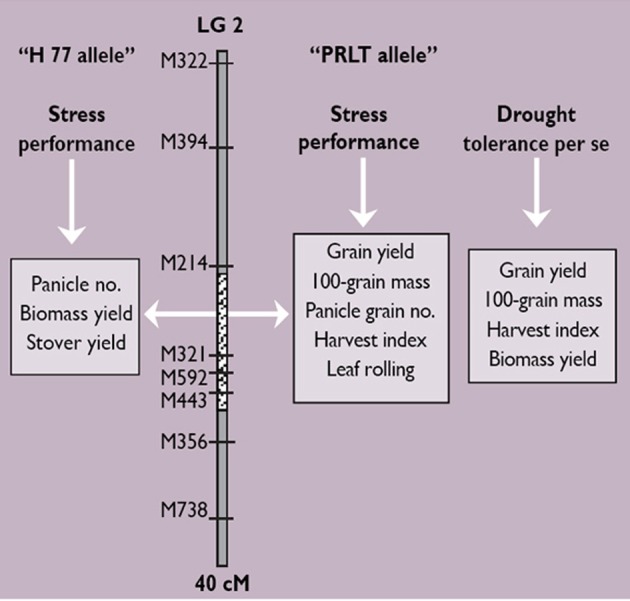
**The outcome of QTL mapping: identification of a set of QTLs for different traits (leaf rolling, biomass yield, dry straw yield, panicle number, panicle grain number, 100-grain mass, grain yield, and harvest index) in pearl millet in three drought nursery experiments**.

For terminal drought tolerance, contrasting parents PRLT 2/89-33 (tolerant) and H77/833-2 (sensitive) were identified and crossed. Then, selfing was done for two generations. Test crossing was done on F_4_:F_2_ derived progenies, using several pollinators, and measuring the GCA for PNHI (Figure 2). In doing this, two parents and 19 product lines all combined to five different testers were used, giving 105 Drought Tolerance QTL-near isogenic line (NIL) testcross hybrids. These materials were evaluated during the summers of 2003 and 2004 in the drought nursery at ICRISAT-Patancheru under three moisture regimes (fully irrigated conditions; early stress imposed by stopping irrigation at booting; late stress imposed by stopping irrigation at flowering). The experimental design is an alpha design with two-row plots and 4 m rows, into three replications. Usually, many QTLs are identified, each differing in the percentage of the variation in phenotypic data that they explain. QTLs can be identified for many different traits, some of these collocating at the same portion of the chromosome (Figure 3).

**Figure 4:**
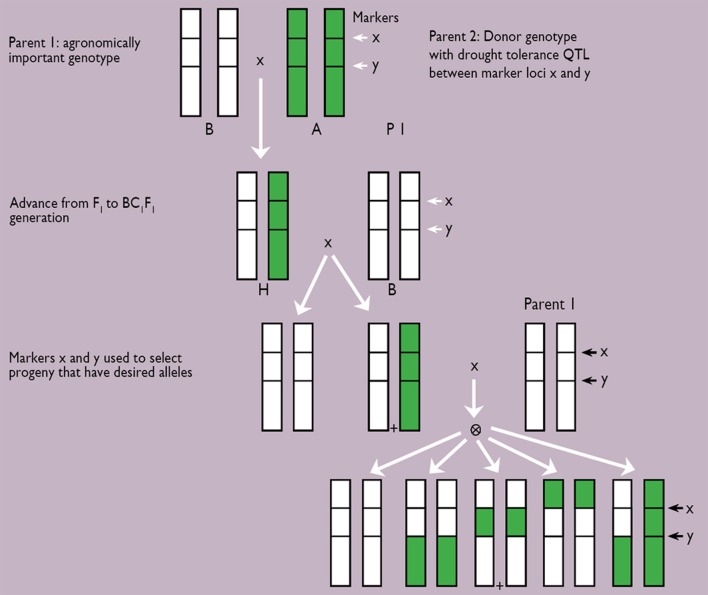
**Scheme for marker-assisted backcrossing of desired (+) segregants**. A QTL was introgressed after four rounds of marker-assisted backcrossing (Source: Hash, unpublished).

The likelihood of odds (LOD) score assesses, in part, the importance of a QTL. The higher the LOD score, the more significant is the QTL. Among the many usually identified, one or two major QTLs are chosen to be introgressed into a genetic background of either elite germplasm, or locally adapted germplasm. A few rounds of backcrosses are usually needed to end up with introgression lines having maintained most, if not all, of the recurrent parent genome, except for the portion flanked by the marker pair (Figure 4). Results of the whole effort are represented in Figure 1, where the introgression of a major QTL for terminal drought tolerance from donor parent PRLT/89-33 in the background of sensitive parent, high tillering H77/833-2, led to panicles with a higher percentage of seed setting, and a high 100-grain weight. The output is a genotype that looks essentially like the recurrent parent but with a higher threshing index of the panicle.

### Trial planning

The creation and genotyping of mapping populations is often the most expensive part of the overall effort, but its ultimate success depends on the effectiveness of the phenotyping procedure in detecting repeatable, highly heritable differences among recombinant lines, that permit the identification of robust QTLs. Drought is a particularly difficult topic for molecular mapping, because it is not possible to define or measure tolerance with the same clarity or precision as disease resistance or morphological or physiological traits. Nor is it easy to manage experimental drought or saline environments with a high level of control and repeatability. One key aspect in the implementation of a phenotyping experiment is to carefully exclude any possibility of non-genetic variation in the measured traits. Therefore, extra effort is needed in the conceptualization, design and management of phenotyping programmes for drought, to maximize the chances of identifying highly contrasting materials and, further, QTLs that will be useful in the future improvement of tolerance in the target crop and in the target environment.

### Water stress management and characterisation

**Figure 5:**
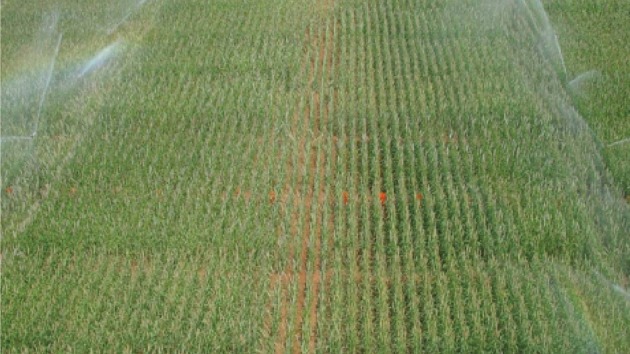
**Typical line source experiment, which allows the imposition of a gradient of watering regimes, from fully irrigated conditions close to the irrigation line, to severely stressed at the point most distant from the line (Source: SMH Rizvi, unpublished)**.

Pearl millet is usually grown in areas receiving less than 500 mm of rainfall annually. It is usually planted at the start of the rains, either in the Sahelian areas or the arid semi-desert areas of northeast India (Rajasthan) and southwest Pakistan (Bidinger et al., 1987a,b). Because the duration of the rains is normally shorter than the duration of the crop, the stress that millet commonly experience is a terminal drought, whereby seed filling occurs with plants depending on the moisture available in the soil. We have previously seen that the phenotyping of terminal drought tolerance uses three water regimes: full irrigation; early stress imposed by stopping irrigation at booting; and late stress imposed by stopping irrigation at flowering (Serraj et al., 2005). However, the intensity of stress imposed is also very important, and certain genotypes can react differently to different intensities. Therefore, line source treatments have also been set up. These are based on the fact that sprinkler irrigation provides a decreasing supply of water when moving away from the sprinkler head, and this decline is roughly linear (unpublished). This allows imposition of a gradient of irrigation and, therefore, a gradient of stress intensities. This approach (Figure 5) has been used to assess a limited number of promising drought tolerant QTL–NIL test-cross hybrids.

#### Protocol and measures

The DRI is a measure of tolerance to terminal drought conditions, as explained previously. Experiments have been conducted to expose pearl millet to a range of environments and different intensities of stress from flowering onwards. In each of the treatments, the yield components (grain number per panicle and surface area, grain size) are measured, as well as time to flowering and to maturity. To separate out the effects of yield potential and phenology from the yield under stress to obtain the DRI, the following equation is used:

Ys=aYc+bFl+cDRI+E

DRI is usually well correlated to PNHI and, therefore, PNHI is routinely calculated from the yield components.

### Plant water strategy

Accurate field or controlled environment phenotyping of germplasm accessions or mapping populations for traits as complex as drought tolerance is almost certainly the limiting factor in our ability to detect contrasting materials and to discover molecular markers for such traits. The PNHI trait remains complex, and its measurement under field conditions remains subject to field variability and the usual experimental errors associated with field evaluations. For that reason, secondary traits that correlate well with field performance and that can be measured under more controlled conditions are very useful. Several hypotheses can explain differences in the PNHI. Better grain filling during the post-anthesis period could be due to water saving in the soil profile from the time it is wet. The water saved would then be available later on for grain filling. Another possibility is that deeper or more profuse rooting would allow the crop to sustain water uptake and continue grain filling in the latest part of the grain-filling period. Other hypotheses to explain differences in PNHI and, eventually, differences in grain yield under terminal drought stress can be formulated. Such hypotheses lead to the identification of putative secondary traits.

### Phenotyping traits

The hypotheses above are currently being tested. For instance, we have found that the rate of water loss per unit of leaf area and time was lower in PRLT/89-33, our terminal drought tolerant parent and donor for the major drought tolerance QTL on LG2, compared to H77/833-2, a terminal drought sensitive genotype (Vadez et al., 2007). These differences were found under well-watered conditions and were consistently found across experimental seasons, at both the pre-flowering and post-flowering stages. This trait, which appears to be constitutive and also relatively easy to measure, is very suitable for phenotyping the RIL progenies of the cross between PRLT/89-33 and H77/833-2.

We also measured the root depth and root length density in a set of pearl millet genotypes contrasting for terminal drought tolerance, and including PRLT/89-33 and H77/833-2, as well as terminal drought sensitive 841B and tolerant 863B, along with some introgression lines with the DT QTL from PRLT/89-33 in the background of H77/833-2. Root traits were measured under water stress conditions, and all terminal drought tolerant materials appeared to have more profuse rooting in the deep soil layers than did sensitive materials (Vadez et al., 2007). In contrast, there seemed to be little difference under well-watered conditions. Therefore, rooting appears to be an adaptive trait that tolerant pearl millet genotypes “develop” under stress conditions. However, the measurement of rooting was time-consuming and showed fairly large experimental errors. Since the putative role of deeper rooting would be to sustain water uptake during the latest part of the grain-filling period, the phenotyping of root trait differences would better be based on the volume of water uptake during the grain-filling period.

Phenotyping work on pearl millet has, so far, focused on terminal drought tolerance. QTLs have been identified under the screening conditions of the drought nursery at ICRISAT-Patancheru. Soils are heavy and deep Alfisols, with a significant water-holding capacity (well above 200 mm), thereby allowing the secondary traits described above to be relevant under such conditions. A similar situation may also prevail in pearl millet cultivation in certain areas of West and Central Africa endowed with heavy soils. However, the terminal drought tolerance QTL identified under these particular drought conditions may not be suited to other types of drought environment, for example, those prevalent in semi-desert areas such as northwestern India, or in areas of West and Central Africa, where sandy soils with limited moisture availability dominate.

Therefore, it is crucial in a phenotyping exercise to ensure that the traits that would be measured are the traits that are relevant for the target area. In that respect, the past 30 years have taught us a lot with respect to traits for adaptation to terminal drought tolerance. We feel that more needs to be learnt about traits that would contribute to better drought adaptation to harsher environment. Only the improvement of phenotyping capacities in all representative types of stress environment would allow us to understand the specificity of each trait and would improve the accuracy of trait-based marker-assisted breeding for drought.

## Conclusions

Phenotyping remains the foundation for success in every marker-assisted selection approach, particularly for such complex trait as drought. Precise and accurate phenotyping methods have been set up to phenotype the response to terminal drought in pearl millet, using PNHI as a proxy for an increased yield under terminal drought, independently of yield potential and time to flowering. Such precise phenotyping was possible because of the large human and physical investment made in that activity at ICRISAT's Headquarters at Patancheru. This has led to the identification of a major terminal drought tolerance QTL on LG2 of the pearl millet genome. The subsequent introgression of that QTL into the background of a sensitive hybrid, HHB67, has led to an improved version of that hybrid, HHB67-improved. This QTL remains large in size and, therefore, relatively difficult to introgress. Secondary traits for the high PNHI of the terminal drought tolerant lines would be needed to refine the QTL interval and facilitate its use in modern breeding. More work would also be needed to identify the traits involved in better performance and resilience of pearl millet under other types of drought environment. This would require significant investment in human and physical capacity to phenotype in these other environments for modern breeding to be used.

### Conflict of interest statement

The authors declare that the research was conducted in the absence of any commercial or financial relationships that could be construed as a potential conflict of interest.
